# Maternal Diet Alters Trained Immunity in the Pathogenesis of Pediatric NAFLD

**DOI:** 10.33696/immunology.2.061

**Published:** 2020

**Authors:** Karen R. Jonscher, Jesse Abrams, Jacob E. Friedman

**Affiliations:** 1 Department of Biochemistry and Molecular Biology, University of Oklahoma Health Sciences Center, USA; 2 Harold Hamm Diabetes Center, University of Oklahoma Health Sciences Center, USA; 3 Departments of Physiology and Pediatrics, University of Oklahoma Health Sciences Center, USA

**Keywords:** Pediatric NAFLD, Trained immunity, Pioneering bacteria, Microbiome, Epigenetic reprogramming, Hematopoietic stem cells

## Abstract

Pediatric nonalcoholic fatty liver disease (NAFLD) affects 1 in 10 children in the US, increases risk of cirrhosis and transplantation in early adulthood, and shortens lifespan, even after transplantation. Exposure to maternal obesity and/or a diet high in fat, sugar and cholesterol is strongly associated with development of NAFLD in offspring. However, mechanisms by which “priming” of the immune system in early life increases susceptibility to NAFLD are poorly understood. Recent studies have focused on the role “non-reparative” macrophages play in accelerating inflammatory signals promoting fibrogenesis. In this Commentary, we review evidence that the pioneering gut bacteria colonizing the infant intestinal tract remodel the naïve immune system in the offspring. Epigenetic changes in hematopoietic stem and progenitor cells, induced by exposure to an obesogenic diet *in utero*, may skew lineage commitment of myeloid cells during gestation. Further, microbial dysbiosis in neonatal life contributes to training innate immune cell responsiveness in the gut, bone marrow, and liver, leading to developmental programming of pediatric NAFLD. Comprehensive understanding of how different gut bacteria and their byproducts shape development of the early innate immune system and microbiome will uncover early interventions to prevent NAFLD pathophysiology.

## Pediatric NAFLD and Why It Is a Pressing Issue

Nonalcoholic fatty liver disease (NAFLD), a spectrum of pathologies ranging from simple steatosis to fibrosis and cirrhosis, is the most common cause of chronic liver disease, affecting over 80% of adults with obesity [1], one third of obese children ages 3–18 in North America [2] and ~10% of the general pediatric population [2]. NAFLD in children progresses more rapidly than in adults [3–7], often leading to cirrhosis and transplantation in early adulthood [6]. Half of children presenting with NAFLD have already progressed to the more serious form of nonalcoholic steatohepatitis (NASH) at time of diagnosis [2,8,9] and their survival, even after transplantation, is shortened when compared with the general population [5]. Maternal obesity is a significant risk factor for pediatric NAFLD [10,11]. However, a major limitation in this field is the lack of fundamental understanding as to how maternal diet and/or obesity sets liver physiology and development of the immune system, beginning early in life, on a course toward NAFLD.

NASH is characterized by inflammation, oxidative stress, mitochondrial dysfunction, elevated levels of pro-inflammatory cytokines and fibrosis. Data from our studies in a nonhuman primate model of maternal obesity [10,12–18], combined with findings from other studies in mice [19] and humans [20], indicate that risk factors for NAFLD begin *in utero*, altering tissue function at the cellular and molecular levels. Persistence of liver steatosis and inflammation in juvenile animals switched to a healthy diet at weaning suggests that developmental changes have permanent epigenetic effects which alter metabolic outcomes and increase vulnerability to accelerated fibrosis in offspring [10,11,21]. DNA methylation, covalent modification of histones, and the expression of non-coding RNA are epigenetic phenomena found in livers from children [22–25], adults [26–33], and rodents [34–39] with NAFLD (reviewed recently by Campisano, et al.[40]). Still, insights into the mechanisms by which maternal diet and obesity prime the immune system toward inflammation and liver damage are lacking.

In NAFLD, portal infiltration of macrophages is an early event predicting disease progression, and occurs in the steatotic liver before inflammation or fibrosis develops [41]. Hepatic inflammation is driven, in part, by activated endogenous liver macrophages (Kupffer cells), innate immune cells arising from fetal liver, and from infiltrating monocyte/macrophages arising from bone marrow precursors [42,43]. Kupffer cells and monocyte/ macrophages can either promote hepatic inflammation and fibrosis (M1-like, pro-inflammatory) [10,44–54], or resolve inflammation and prevent progression to fibrosis (M2-like, pro-restorative/reparative) [55]. In children with NASH, numerous activated macrophages are found in the spaces between damaged hepatocytes [56]. We (and others) have shown that mice exposed to a maternal “Western-style” diet (WD) have increased pro-inflammatory macrophage activation in the liver and accelerated hepatic fibrosis as adults [57]. Further, our published data in a nonhuman primate model demonstrated increased expression of pro-inflammatory cytokines (*IL1B* and *TNFA*) in hepatic macrophages isolated from 1-year-old offspring born to WD-fed mothers and weaned to a chow diet [14]. Whether maternal diets during gestation or lactation alter the fate of developing macrophage precursors remains an important unanswered question.

## Gut Microbial Dysbiosis in Early Life Influences the Developing Immune System

Evidence suggests maternal and postnatal factors that impact the developing infant gut microbiome include maternal diet [58], infant diet in early life [59], antibiotic use, and delivery by Caesarean section [60,61]. Studies in animals [62–65] and neonates [66,67] demonstrate that “pioneering” gut bacteria in early life profoundly shape development of the innate and adaptive branches of the immune system, with persistent effects on immune function later in life [68,69]. Because both the gut microbiome and gut immune cells develop and mature during the neonatal period [70,71], even a brief disruption to the microbial community structure during this window can induce immunological changes that persist into adulthood [69]. For example, Olszak, et al. showed colonizing neonatal - but not adult - germ-free mice with pioneering microbes protects from immune cell-mediated pathology in adulthood [69]. Together, these studies demonstrate the critical importance of initial colonizers to the gut microbiome – immunity axis [72].

Most microbiota-driven immune alterations are assumed to be postnatal effects induced by the neonate’s own microbiota [73–75]. However, detection of bacteria in the placenta, amniotic fluid, and meconium suggests the possibility of fetal colonization during gestation, which alters development of the naïve fetal innate immune system [76] and impacts maturation of the hematopoietic system [77]. Gomez de Agüero, et al. showed this by transiently colonizing germ-free pregnant dams with *Escherischia coli*, which led to enhanced ILC3 and F4/80^+^ CD11c^+^ mononuclear cell populations in the gut of neonates, reprogrammed intestinal transcriptional profiles, and increased expression of genes involved in metabolism, oxidative stress, and innate immunity [78]. Moreover, bacterial colonization in early life influences immune development in primary lymphoid organs beyond the gut, including in the bone marrow [77]. Gut microbial and non-microbial ligands, including short-chain fatty acids (SCFA) and bile acids, induce a memory response in innate cells mediated by pattern recognition receptors (PRRs), which recognize microbe- or pathogen-associated molecular patterns (MAMPs or PAMPs) [79]. Pattern recognition receptors include families of Toll-like receptors (TLRs) and nucleotide-binding oligomerization domain (NOD)–like receptors (NLRs). Microbe- and pathogen-associated molecular pattern recognition via these PRRs affects differentiation and function of myeloid and lymphoid lineage immune cells [80–82], inducing both myeloid bias in long-term hematopoietic stem cells within the bone marrow [83] and immune memory in differentiated descendents [84]. Lipopolysaccharide (LPS), a canonical ligand for TLR, dose- and duration-dependently induces tolerance or potentiates innate immune memory [85]. LPS isolated from *E. coli* has immunostimulatory activity leading to endotoxin tolerance, which is inhibited by LPS from *Bacteroides* [86], supporting the hypothesis that in early life, the abundance ratio of Enterobacteriaceae to other commensals critically regulates immune development and health in later life.

In addition to bacteria, bacteria-derived dietary metabolites transfer from mother to fetus, affecting immune development. In mice, feeding pregnant dams dietary fiber or acetate alone protected offspring from allergic airway inflammation [87] and maternally-derived SCFAs played a role in Foxp3^+^ regulatory T cell generation in the neonatal thymus [88]. Maternal gut bacterial products other than SCFAs may have effects on programming immunity in the offspring. Metabolites including taurine, polyamines, retinoic acid, and indoles (byproducts of tryptophan catabolism) have roles in maintaining immune homeostasis, gut barrier integrity, and arginine levels, as well as regulating the inflammasome [89].

Enterobacteriaceae are an important family of Gammaproteobacteria, a class of aerobic, LPS-producing pioneering microbes that are abundant in the stool from full-term, vaginally-delivered newborns [90]. *E. coli* and *Enterobacter*, facultative anaerobic pioneering bacteria, rapidly grow and metabolize lactose from breast milk to acetate and other SCFAs and create a reduced acidic environment. This environment is favorable for later colonization by slower-growing, anaerobic acidophiles such as *Bacteroides, Bifidobacterium,* and *Clostridium* [91,92], which consume most of the available sugar and excrete large amounts of acetate and other SCFAs [93]. Unlike in adults, early exposure to Enterobacteriaceae in newborn rodents and humans provides LPS-driven inflammatory challenges important for training or “priming” the early immune system, and protecting against excessive inflammatory, autoimmune, and metabolic disorders later in life. LPS/endotoxin binds to receptors on innate immune cells, including TLR4, and modulates the host innate immune response though mechanisms such as endotoxin tolerance or trained immunity [94–96]. Subtypes derived from specific *Bacteroides* species exhibit lower endotoxicity than LPS isolated from other enteric bacteria [97]. Immune sequelae linked to early life dysregulation of the Enterobacteriaceae to Bacteroidetes balance were recently described in human infants. Vatanen, et al. elegantly showed *Bacteroides* species, in the microbiota of infants from countries with high susceptibility to allergies and type 1 diabetes (T1D), produced an LPS subtype that inhibited immunostimulatory activity of *E. coli* LPS *in vitro*, compared with those infants colonized predominantly by Enterobacteriaceae. *In vivo*, intraperitoneal injection of *E. coli*-derived LPS led to endotoxin tolerance in immune cells and delayed onset of T1D in a mouse model, whereas LPS from *Bacteroides* was not protective [86].

The impact of maternal obesity on pioneering bacteria in infants was studied by Lemas, et al. [98] who found reduced abundance of Gammaproteobacteria in stool from 2-week-old infants born to obese mothers, when compared with microbiota from infants born to normal weight mothers [99]. Soderborg, et al. colonized germ-free mice with microbes from infants born to obese mothers, and these mice, when challenged with a short-term postnatal WD, exhibited elevated markers of inflammation and endoplasmic reticulum stress in liver, as well as accelerated obesity and NAFLD. Moreover, these animals exhibited dampened LPS-induced inflammation in bone marrow-derived macrophages (BMDMs) and impaired phagocytosis [99]. A unique feature of pediatric NAFLD is the predilection for children to deposit fat and develop inflammation in the periportal region vs. the more classic perivenular distribution seen in adults [4,100]. This difference is poorly understood, but clinically relevant because periportal inflammation is associated with advanced liver disease [101]. Germ-free mice colonized with microbiota from infants exposed to maternal obesity showed histological evidence for increased periportal inflammation, even while consuming a control chow diet [99]. These provocative findings suggest a mechanistic role for the early life gut microbiota in priming innate immune dysfunction prior to the development of childhood obesity.

## Dietary Exposures and Epigenetic Rewiring Skew Hematopoiesis to Promote Chronic Inflammation

The fetal liver is the major hematopoietic organ of the developing immune system. During gestation, the fetal liver is seeded with monocytes, which are progenitors of liver resident Kupffer cells [102,103], and hematopoietic stem and progenitor cells (HSPCs) which migrate to the bone marrow, where most stay for the remainder of life [104]. HSPCs in the bone are capable of producing all blood cells of the lymphoid (adaptive immune) and myeloid (innate immune, erythroid, platelets) lineages, including monocytes and their macrophage descendants. HSPC-derived monocytes in the fetal liver give rise to Kupffer cells, which maintain self-renewing capabilities throughout life [102,105]. By contrast, infiltrating tissue macrophages differentiate from monocytes that are continuously generated from HSPCs in the marrow and recruited to the liver by damage signals (monocyte/macrophages) [106]. Diet-induced gut microbial dysbiosis shapes development and function of the immune system, in part by regulating the differentiation of HSPCs [107]. Intriguingly, maternally-derived HSPCs have been detected in cord blood [108]. Whether these maternal cells, programmed by a poor diet, seed the fetal bone marrow and educate the developing immune system, or whether an obesogenic maternal diet and resultant gut microbial dysbiosis directly program neonatal HSPCs to promote NAFLD are questions warranting further research.

Myeloid cells (monocytes/macrophages), innate lymphoid cells (including NK cells), and bone marrow progenitors [109] exhibit innate immune memory. This memory involves epigenetic rewiring after an initial inflammatory insult and a rapid, non-specific enhanced response to subsequent exposures [109]. Microbial signals, including peptidoglycans [110], Bacillus Calmette-Guérin [111], and β-glucan [83], alter epigenetic modifications in HSPCs, induce long-lasting changes in cellular lineages, and stimulate inflammatory priming of differentiated myeloid cells [77]. The induced memory may persist from weeks to months [112,113]. In fetal mice, exposure to maternal WD remodels fetal liver HSPCs to exacerbate the inflammatory immune response, skew commitment to the myeloid lineage, and favor differentiation at the expense of self-renewal [114]. Later-life consequences of this early adaptation can be observed in adult WD-fed mice, where HSPCs are biased toward the myeloid lineage and generate a large pool of pro-inflammatory cells [115]. Moreover, in response to short-term post-natal WD exposure, Christ, et al. found upregulation of genes involved in cellular proliferation, skewing of granulocyte-monocyte progenitors toward the monocytic cell lineage, and increased availability of enhancer regions (including TLR4) [116].

In our mouse model of maternal WD, we showed elevated fumarate in liver macrophages and BMDMs from adult offspring of obese pregnancy [117]. This metabolic change triggers epigenetic remodeling toward a pro-inflammatory phenotype. Innate immune memory is characterized by a potentiated response to inflammatory stimuli, accompanied by a metabolic shift to aerobic glycolysis and a dysfunctional TCA cycle, termed “metabolic reprogramming” [118]. Metabolic and epigenetic reprogramming occurs in myeloid cells [119,120], including HSPCs [83] and their descendants, and alters transcription of genes in inflammatory, immune, and metabolic pathways [116]. TCA cycle intermediates, such as succinate and fumarate, promote expression of genes supporting the pro-inflammatory (M1-like) macrophage phenotype through stabilization of hypoxia-inducible factor-1α [121–123] and histone acetylation of glycolytic enzyme genes, including hexokinase 2 and lactate dehydrogenase [124]. Moreover, α-ketoglutarate increases expression of genes promoting a reparative (M2-like) phenotype through epigenetic regulation [125,126].

Finally, Wang, et al. showed that DNA hypermethylation at the peroxisome proliferator-activated receptor γ1 promoter in adipose tissue macrophages suppressed the ability of these cells to adopt an alternatively activated, reparative phenotype (characterized by elevated “M2” markers such as ARG1, MRC1, and CLEC10A). Moreover, failure to adopt an M2-like phenotype was associated with weight gain and insulin resistance in mice fed a chronic high-fat diet [127]. These studies suggest that HSPC immunometabolism, even in early life, contributes to programming adult metabolic disease when dysregulated by exposure to an obesogenic maternal diet. It will be important for future work to determine whether similar mechanisms act in bone marrow and the liver.

In conclusion, pediatric NAFLD is a growing problem worldwide with a complex pathophysiology. Therefore, it is critical to elucidate factors driving development of the neonatal immune system, particularly in bone marrow and liver, to determine how maternal obesity alters infant immunity and drives development of pediatric NAFLD. Mechanistic studies, including perinatal maternal or infant supplementation with specific bacteria, could identify bacterial strains or metabolic functions responsible for pro-inflammatory priming of the early innate immune system. Going forward, it will be important to advance our knowledge on how the immune system senses changes in early dietary metabolite composition to either initiate an appropriate inflammatory response or promote inflammatory pathophysiology, such as NAFLD. We must 1) determine whether dietary metabolites other than maternal SCFAs are transferred to the fetus *in utero*, or to offspring in early life, and characterize metabolite effects on development of the immune system in the fetal liver and/or offspring bone marrow; and 2) evaluate the impact of maternal metabolites on colonization and development of the infant intestinal microbiota and test if these early bacteria exert life-long epigenetic changes in HSPCs and their macrophage descendants, accelerating disease pathophysiology. Comprehensive understanding of how maternal diet and obesity influence development of the innate immune system continues to be a major challenge. But, hopefully, this work will lead to early interventions to prevent numerous metabolic diseases associated with inflammation, including obesity, cardiovascular disease, and NAFLD pathophysiology in children.

## Figures and Tables

**Figure 1:**
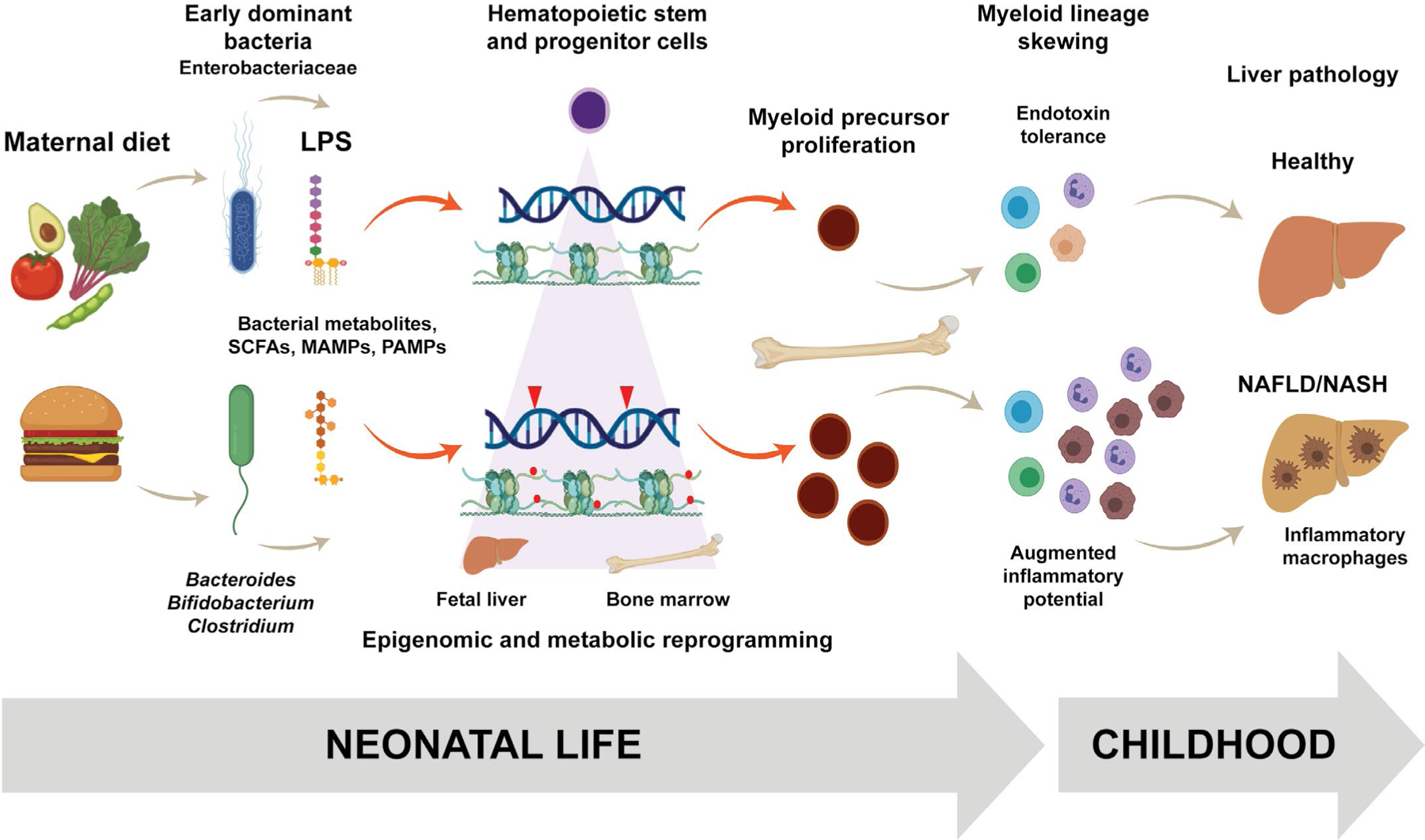
Early pioneering bacteria induce metabolic reprogramming of innate immunity and increase susceptibility to pediatric NAFLD in offspring. Maternal Western-style diet, high in fat, sugar, and cholesterol, alters initial bacterial colonization in infants. By reshaping the ratio of dominant bacterial species in the gut during early life, the composition of the lipopolysaccharide (LPS) pool, bacterial metabolites, short-chain fatty acids (SCFA), microbe-associated molecular patterns (MAMPs) and pathogen-associated molecular patterns (PAMPs) are also varied. These altered microbial signals induce expansion of hematopoietic stem and progenitor cells, myeloid lineage skewing, and inflammatory polarization of monocyte/macrophages recruited to the steatotic liver. Therefore, a shifted ratio of Enterobacteriaceae to acidophilic commensals, such as *Bacteroides* or *Bifidobacterium*, may mediate susceptibility to NAFLD in childhood and accelerate disease progression through “training” of innate immune cells. This figure was generated with the assistance of BioRender (www.biorender.com).
